# Sustainable manufacture of insect repellents derived from *Nepeta cataria*

**DOI:** 10.1038/s41598-017-18141-z

**Published:** 2018-02-02

**Authors:** Gregory S. Patience, Ginette Karirekinyana, Federico Galli, Nicolas A. Patience, Cariton Kubwabo, Guy Collin, Jean Claude Bizimana, Daria C. Boffito

**Affiliations:** 1 Département de Génie Chimique, École Polytechnique de Montréal, 2900, boul. Édouard-Montpetit, H3C 3A7 Montréal, QC Canada; 2 Agence consultative en éthique de coopération internationale (ACECI), 11 Rue Mugamba, Quartier Rohero II, Bujumbura, Burundi; 3 Département des sciences fondamentales, UQAC, G7H 2B1 Saguenay, QC Canada; 4 Università degli Studi di Milano, Dipartimento di Chimica, via Golgi 19, 20133 Milano, Italy; 5 Department of Bioresource Engineering, McGill University, 3610 University Street, H3A 2B2 Montreal, Canada

## Abstract

Malaria devastates sub-Saharan Africa; the World Health Organization (WHO) estimates that 212 million people contract malaria annually and that the plasmodium virus will kill 419 000 in 2017. The disease affects rural populations who have the least economic means to fight it. Impregnated mosquito nets have reduced the mortality rate but the *Anopheles* mosquitoes are changing their feeding patterns and have become more active at dusk and early morning rather than after 22h00 as an adaptation to the nets. Everyone is susceptible to the *Anopheles* at these times but infants and pregnant women are the most vulnerable to the disease. Plant-based mosquito repellents are as effective as synthetic repellents that protect people from bites. They are sustainable preventative measures against malaria not only because of their efficacy but because the local population can produce and distribute them, which represents a source of economic growth for rural areas. Here, we extract and test the essential oil nepetalactone from *Nepeta cataria* via steam distillation. Families in endemic areas of Burundi found them effective against bites but commented that the odor was pungent. An epidemiological study is required to establish its clinical efficacy.

## Introduction

Malaria is a leading cause of human mortality. In 2015, the World Health Organization (WHO)^1^ estimated that 212 million cases of malaria occurred, leading to 419 000 deaths – 82% of casualties are in sub-Saharan Africa. Children under the age of 5 account for 64% of victims. Malaria’s mortality rate dropped by 31% between 2010 and 2015 in Africa and the life expectancy of children under 5 increased by 1.2 years. However, in 80% of malaria endemic countries, mosquitos developed resistance to at least one insecticide. Furthermore, in Burundi, deaths have increased by 13% in the last year^2^, stunting economic growth^3^ where the annual per capita income is lower than 400 USD^4^ and farmers hire labour at 1 USD a day. As with the mass drug administration of chloroquine, which contributed to the resurgence of malaria in Peru after 30 years of low incidence^5^, *Plasmodium falciparum* protozoa may develop resistance to artemisinin monotherapy^6^. Mutations of the K13 gene are markers of artemisinin resistance but tetraoxane-based compounds have inhibitory characteristics against various strains of the protozoa^7^. Artesunate is more effective than quinine with fewer side effects but a child administered a high dose rectally died due to its toxicity^8^. Moreover, Burke *et al*.^9^ reported that *Anopheles vaneedeni* became a new malaria vector complicating the fight against the disease.

Preventing rather than treating the disease is a better approach; sleeping under nets impregnated with insecticides has reduced the world mortality rate from 2 million a year; they save 5.5 per 1000 children yearly^10^. Insecticides are losing their efficacy due to increasing mosquito resistance^11^. Furthermore, weather patterns like La Niña can cause unexpected peaks in mosquito populations^12^. Graves and Gelband^13^ tested SPf66, CS-NANP, RTS,S, MSP/RESA vaccines and their ability to prevent different stages of malaria. Theoretically, SPf66 and MSP/RESA protect against the asexual stages of plasmodium parasites whereas the other two target the sporozoite stages. CS-NANP and MSP/RESA offered no protection against malaria, SPf66 was ineffective and RTS,S reduced malaria episodes by 58%. Unfortunately, vaccines’ mass production and distribution is too expensive for sub-Saharan Africa^14^. Furthermore, the mosquitos are changing their feeding patterns to adapt to the mosquito nets. Moiroux *et al*.^15^ demonstrated that mosquitos have adapted to insecticide treated nets by changing their biting habits. Proportion of outdoor biting increased from 45% to 68%^16^. Thus, nets will be less effective for young children and pregnant women, who are the most vulnerable to the disease.

Cutaneous mosquito repellents (MR) are one means to reduce the frequency of mosquito bites. N,N-diethyl-meta-toluamide (DEET) is an effective mosquito repellant^17–20^ but is cost prohibitive for rural sub-Saharan populations. Targeting indigenous plants as a source of mosquito repellents will stimulate local economies and at the same time protect the population. Odalo *et al*.^21^ extracted and tested the topical repellency of essential oils indigenous to Kenya from *Conyza newii (Compositae)*, *Plectranthus marrubioides (Lamiaceae)*, *Lippia javanica (Verbenaceae)*, *Tetradenia riparia (Lamiaceae)*, as well as *Tarchonanthus camphoratus (Asteraceae)*. The oils, under their experimental conditions (3 min, forearm exposure), repelled mosquitoes better than DEET. *N. cataria*, commonly known as catnip or catmint, is a species of the Lamiaceae family. It is native to temperate and tropical zones in Asia and in Europe and is widely cultivated^22^. *Nepeta cataria* (*N. cataria*) has many uses in traditional medicine including treatment of chills, colds, constipation, headaches, infections, inflammations, rheumatism, sore throats, spasms, and stomach aches^23^. *N. cataria*’s essential oil possesses antibacterial and antimicrobial properties^24,25^. Nepetalactone (NPL) is the major constituent of this oil^26–29^. DEET, applied to the forearm, repelled *Aedes aegypti*, *Anopheles quadrimaculatus*, and *Anopheles albimanus* for 426 min, 96 min, and 87 min, respectively^29^. A 25% volume fraction of DEET in ethanol repelled *Aedes aegypti* for 8 h^30^. Whereas, Bernier found that a dose of 0.5 mg cm^−2^ of DEET applied to a muslin cloth patch was active for 24 h. Catnip’s oil is a better spatial repellent than DEET and demonstrated effective topical repellency properties^31^.

NPL is as active as DEET and its hydrogenated form – dihydronepetalactone – is two times more active than DEET when formulated with isopropyl alcohol (1% w/v)^32^. Among 41 different essential oils applied to skin, Catnip’s offered protection for 480 min^33^. Moreover, *N. cataria* exhibits a more favorable safety profile than DEET^34^. Local communities widely accept essential oil based MR. In Ghana, 97% of the studied population desired to continue applying the MR after the 3-month trial^35^. Mng’ong’o *et al*.^36^ found that six repellent plants essential oil were widely accepted by the population studied due to their efficacy.

Introducing plant based mosquito repellents to vulnerable populations faces economic challenges but also societal and governmental hurdles. Furthermore, they must be nontoxic with respect to dermal and eye contact, ingestion, and inhalation. Unlike synthetic repellents, the chemical composition of essential oils, like *N. cataria*, contain dozens of compounds (Supplemental information S1). The active ingredient must demonstrate its efficacy versus alternatives but they must also be stable with respect to UV exposure, oxidation and high temperature (>40 °C). Moreover, to achieve the largest possible distribution at the lowest cost, local populations must grow, harvest and extract the active ingredients. Birkett and Pickett discovered the efficacy of nepetolactone and nepetolactol in repelling aphids. They distilled the catnip with steam and cyclohexane vapors then reduced the oil with NaBH_4_. In their concluding remarks, they emphasize the potential of plants as source of commercialy vailable products^37^. Here we address the challenges of cultivating *N. cataria* in Burundi and developing a topical mosquito repellent. We compare the composition of the essential oil versus those cultivated throughout the world and measure the thermal stability and its stability versus UV. Finally, we demonstrate that the population is ready to test the mosquito repellents formulated with vegetable oil or as Pickering emulsions based on an acceptability study we conducted in a rural and urban region of the country.

## Results

### *Nepeta cataria* production

**Figure 1:**
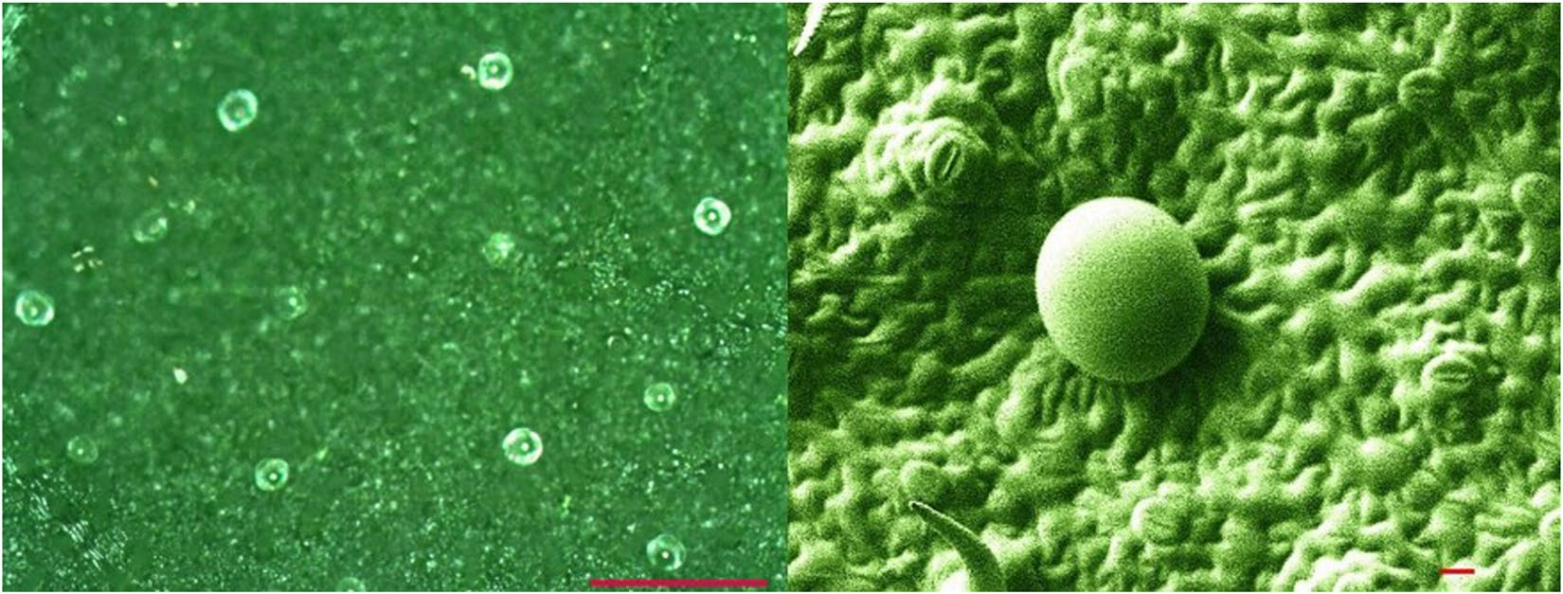
*N*. *cataria* forms glandular trichomes 50 µm in diameter. Leaves sampled at 20 cm from a 40 cm tall plant had 100 trichomes per cm^2^ (10 optical images from 100 mm^2^ samples). Left red marker: 300 µm; right red marker 10 µm.

*N. cataria* (purchased from http://mckenzieseeds.com) is a robust herbaceous short lived perennial plant grown around the globe. Its essential oil is encapsulated in glandular trichomes^38^ measuring 50 µm in diameter (Fig. 1). The plant is cultivated at least three times a year in Burundi and even twice a year in New Jersey where Park *et al*. reported as much as 7.7 t/ha (dry plant matter yield) and an essential oil yield of 12.5 kg/ha^39^. In Burundi, essential oil yield reaches 3 kg/t (dry plant matter), which is double the 1.6 kg/t in New Jersey. The number of trichomes increases until the full flowering stage^40^, while their size remains constant.

### Essential oil characterization

The three principal compounds extracted from *N. cataria* in Burundi were 4a*α*,7α,7aβ-nepetalactone (72%), β-caryophyllene (10%), and *trans*-β-ocimene (4%). The data from this study compare well with other published results describing the composition of catnip oil from various geographical regions around the world. Asgarpanah *et al*.^41^ isolated the essential oils of the *Nepeta* genus and found that 34% of the species contained 4aα,7α,7aα-nepetalactone while 20% (including *N. cataria*) contained the epimer 4aα,7α,7aβ-nepetalactone and 10 plants of 41 contained 4aβ,7α,7aβ-nepetalactone. A GC-MS measured the composition of the essential oil of *N. cataria* grown in Burundi (Table S1, supplementary material). Chalchat and Lamy^42^ did not find nepetalactone in the essential oil isolated from wild catnip *N. cataria L. cv. citriodora* grown in the Drôme region: the major constituents were nerol, geraniol and citronellol. Gilani *et al*.^43^ reported 1,8-cineole, α-humulene (14%) and α-pinene as the 3 major constituents in commercial catnip oil. Other compounds found at relatively higher concentration included α-pinene, limonene and *trans*-β-ocimene. The differences in catnip oil composition (Table 1) are due to extraction techniques, and environmental and agricultural factors like chemotype, soil, growing region, meteorology, pests, drying and extraction methods, etc.

**Table 1:** The main components of *N. cataria* species depends on agricultural practices, soil, age of the plant, collection period, drying, extraction methods, climate and geographic origin (NPL – nepetalactone).

Ref.	Plant matter (Country)	Compound 1	Compound 2	Compound 3
This study	Flowering aerial parts (Burundi)	4aα,7α,7aα-NPL (72.4 %)	β-Caryopyllene (10.2 %)	*trans*-β-Ocimene (3.8 %)
^58^	Aerial parts (USA)	NPL (77.6 %)	Epinepetalactone (15 %)	Caryophyllene (2.8 %)
^59^	Flowering aerial parts (France)	4aα,7α,7aβ-NPL (56.9 %)	Caryophyllene oxide (18.2 %)	β-Caryophyllene (6.2 %)
^60^	Aerial parts, bloom stage (Argentina)	NPL (57.3 %)	Caryophyllene oxide (19.4 %)	β-Caryophyllene (8.1 %)
^61^	Aerial parts (Balkan mountain, Bulgaria)	4aα,7α,7aβ-NPL (78.0 %)	4aβ,7α,7aα-NPL (56.9 %)	Nepetalic acid (1.6 %)
^42^	Flowering aerial parts (France)	Nerol (28.2 %)	Geraniol (27.6 %)	Citronellol (15.1 %)
^62^	Flowering aerial parts (USA)	(*Z,E*)-NPL (54.6 %)	(*E,Z*)-NPL (31.9 %)	β-caryophyllene (11.6 %)
^63^	Flowering aerial parts (Iran)	4aα,7α,7aβ-NPL (28.8 %)	1,8–Cineole (13.5 %)	4aα,7β,7aα-NPL (11.9 %)
^64^	Flowering aerial parts (Turkey)	4aα,7α,7aβ-NPL (70.4 %)	4aα,7α,7aα-NPL (6 %)	4aα,7β,7aα-NPL (2.5 %)
^43^	- (Pakistan)	1,8-Cineole (21 %)	α-Humulene (14.4 %)	α-pinene (10.4 %)
^40^	4-aerial develop-mental stages (Iran)	4aα,7α,7aβ-NPL (55–59 %)	4aα,7β,7aα-NPL (30–31.2 %)	α-Pinene (2.7–4.6%)
^65^	Flowers (UK) *Chemotype A*: *Chemotype B:*	4a*S*,7*S*,7a*R*-NPL (92 %) 4a*S*,7*S*,7a*R*-NPL (17 %)	(4a*S*,7*S*,7a*S*)-NPL (4aS,7S,7aS)-NPL (69.8 %)	(*E*)-(1*R*,9*S*)-Caryophyllene (8 %) (*E*)-(1*R*,9*S*)-Caryophyllene (13.2 %)
^25^	3-aerial develop-mental stages (Iran)	4aα,7α,7aβ-NPL (55–58 %)	4aα,7β,7aα-NPL (30–31.2 %)	α-Pinene(2.7–4.6%)
^66^	Flowering stage (Morocco)	4aα,7α,7aβ-NPL (77.4 %)	Dihydronepetalactone (5 %)	Limonene (4.1 %)
^67^	Aerial parts (Turkey)	NPL (27.5 %)	1,8-Cineole (10.8 %)	Germacrene D (9.2 %)

### Essential oil extraction

**Figure 2:**
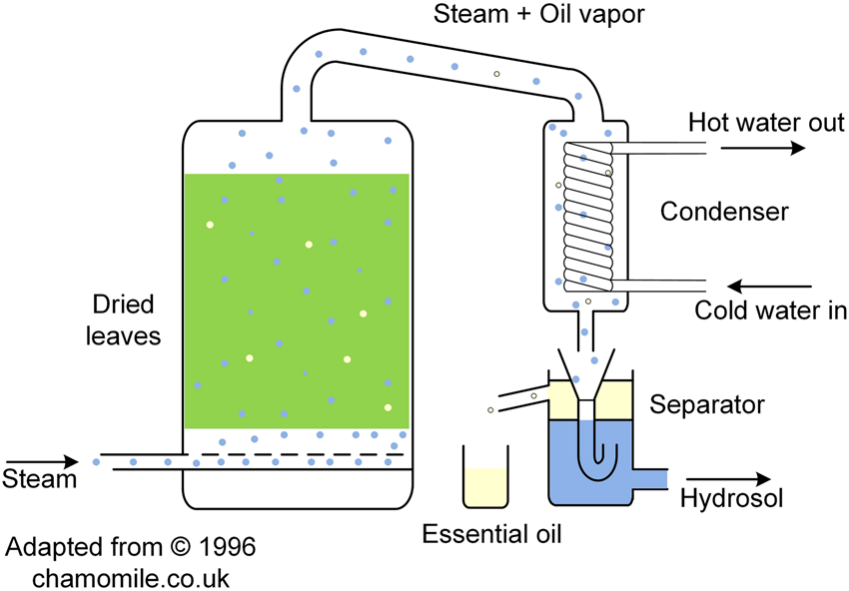
Steam distillation schematic of *Nepeta Cataria* to extract its essential oil.

Batch steam distillation is the standard technology to extract the oil (Fig. 2). However, it is energy intensive since about 1000 kg of water vapour extracts 1 kg of oil. A major program element to develop a process suited for local populations is to identify energy sources.

**Figure 3:**
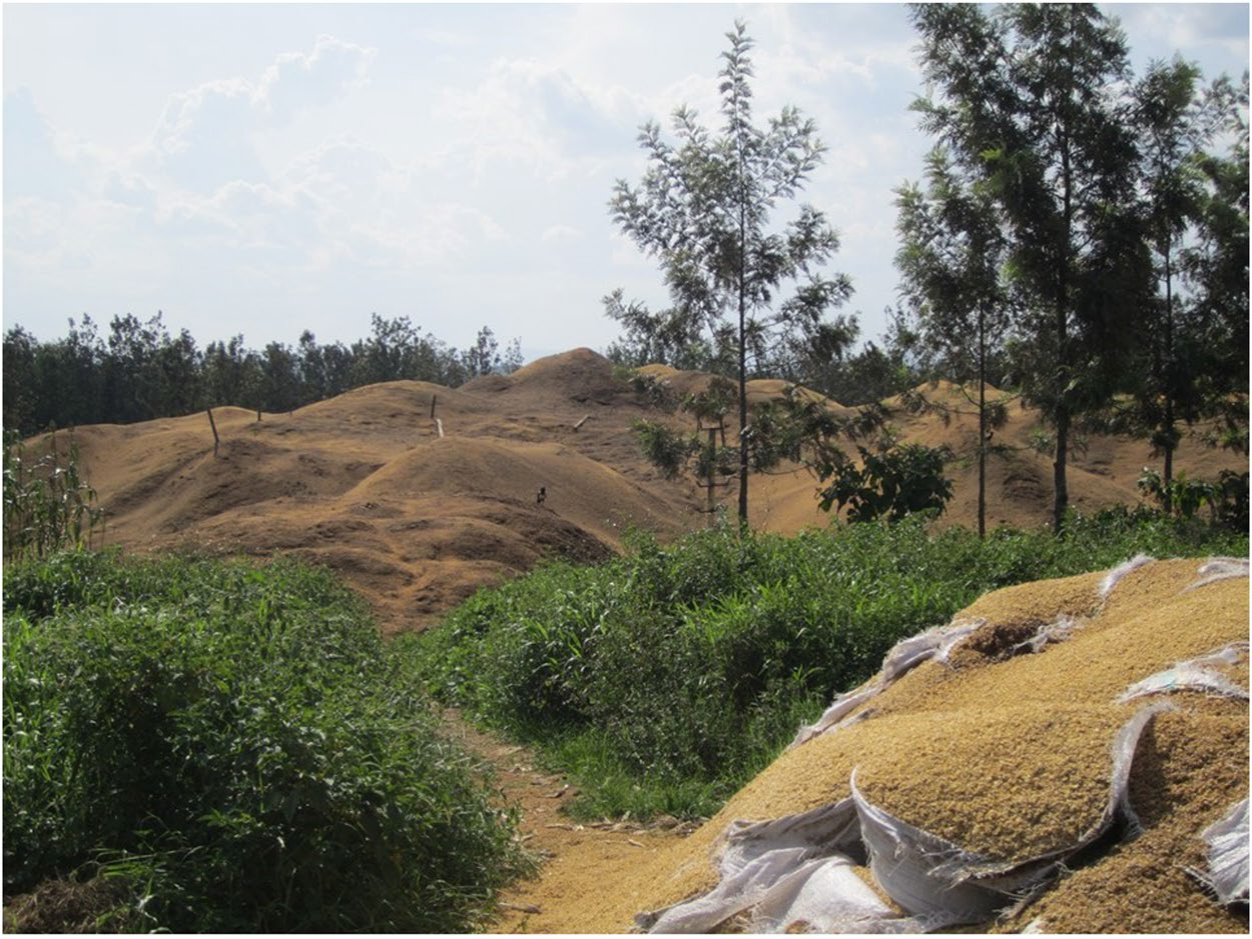
Mounds of coffee husks generated by the SODECO hulling factory, Burundi.

Much of the forests in Burundi, for example, have disappeared as the rural population relies on wood to cook. Alternative energy sources to extract oil include coffee husks (Fig. 3), bagasse from sugar cane, tea, rice husks and other farm residues^44,45^. These lignocellulosic based sources may require torrefaction and pelletization^46^ to increase their energy density. Micro-wave and ultrasound are alternatives to steam distillation with solar energy as the vector to produce electricity. Tests with ultrasound at temperatures from ambient to 60 °C with water and ethanol extracted chlorophyll together with some oil but yields were low. Furthermore, sonication at 20 W (frequency of 20 kHz) destroyed the leaf, which indicates that too much power was applied and that it is not selectively activating the trichomes. Therefore, here we extracted the essential oil with steam distillation.

### Stability study

**Figure 4:**
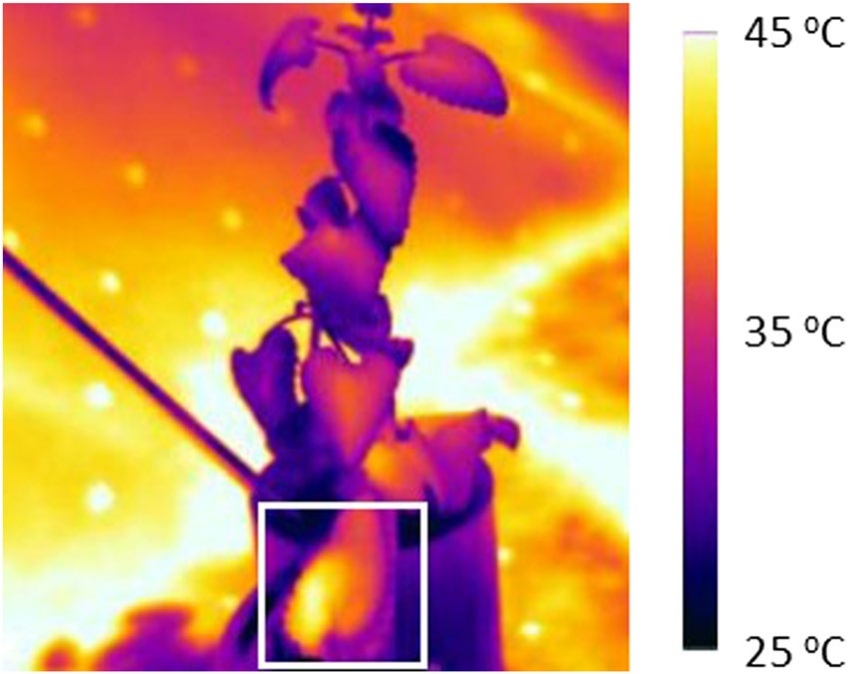
Infrared image of a small branch whose end is immersed in water under the sun at noon (Montréal, July 2017). The ambient temperature was 25 °C. The leaf in the white square was cut from a branch 1 h prior to the photo. The centre of this leaf approaches 45 °C while the edges of the top leaves on the twig are closer to 30 °C.

Producing mosquito repellents locally minimizes transportation, distribution, and storage costs. NPL is susceptible to degradation due to UV, heat and oxygen, which requires additional packaging to reduce light exposure. Average temperatures in north Africa hover well over 30 °C while the average temperature in the central Burundian plateau is 20 °C^47^. Sun radiation heats surface temperatures of inanimate objects well above 50 °C but plant transpiration through stomatal apertures maintains leaves and the trichomes at a lower temperature (Fig. 4). NPL degradation will be most prevalent after the extraction process.

Temperature, light, and oxygen deteriorate essential oils’ integrity and lower the active component concentration. As a bicyclic, monounsaturated terpenoid, nepetalactone undergoes primary oxidation to unstable products, i.e. hydroperoxides, which then convert into stable secondary oxidation products such as alcohols, aldehydes, ketones, epoxides, peroxides, but especially acids^48^. Heat and light promote the cleavage of the unique double bond in nepetalactone by epoxidation or allylic oxidation into alcohols, ketones, and aldehydes^49^. Many of these constituents may be skin irritants, which would reduce their desirability as a topical treatment.

Water is an inexpensive solvent to dilute the active ingredients for cutaneous applications; however, NPL is poorly soluble in water. Blending it with vegetable oil or another solvent increases the overall cost of the lotion. An alternative is to generate a Pickering emulsion stabilized with silica^50^. This may also reduce NPL’s volatility and thereby increase the effectiveness of a single application. We tested nepetalactone stability with respect to temperature at 60 °C and to light with a UV-A lamp at *λ* = 365 nm (Spectroline EA-160) for both pure *N. cataria* oil (7 d) and a 10% *N. cataria* essential oil emulsion over 140 d and measured their iodine and acid values.

**Figure 5:**
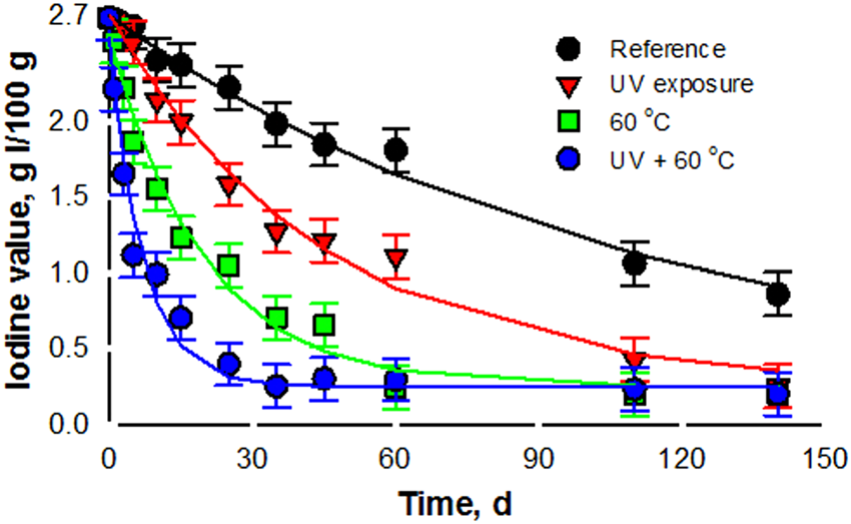
NPL Pickering emulsion stability with respect to IV and 60 °C over 20 weeks. Error bars represent standard deviation, *n* = 3.

Iodine value (IV) measures the number of unsaturations, i.e. both the tendency of a substance to oxidize, as well as the number of unsaturations loss from oxidation, thus being an indirect measure of the essential oil light and heat stability^51^. The IV does not differentiate between primary and secondary oxidation. The IV of the reference oil, preserved from heat and light, was 59 g I_2_/100 g oil. The IV of the *N. cataria* Pickering emulsion before heat and/or light exposure was 2.7 (Fig. 4). Besides the obvious decrease of the IV because of dilution, moisture may saturate double bonds^52^. The IV of the 10% *N. cataria* Pickering emulsion^50^ decreased by 68% in 140 days, whereas the IV of the emulsion exposed to heat and-or light decreased by over 90% in 7 days, indicating a more severe oxidation of the double bonds (Fig. 5).

**Figure 6:**
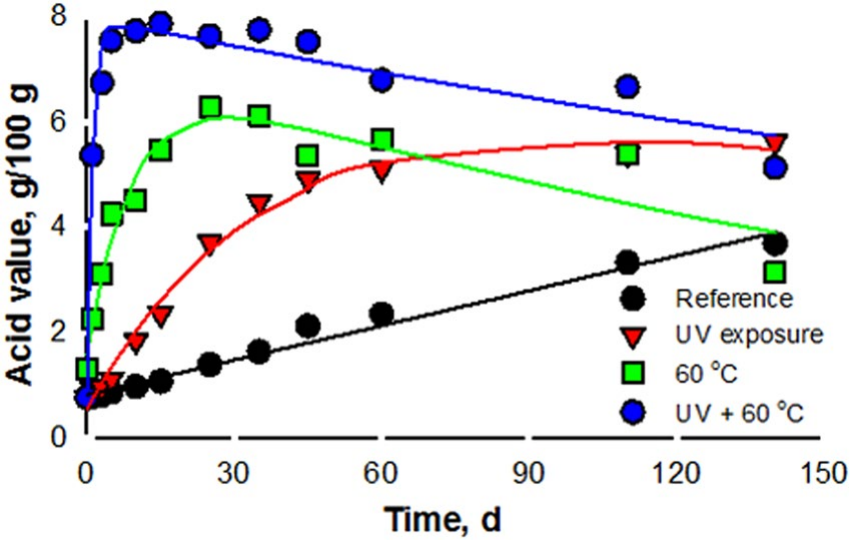
NPL Pickering emulsion stability with respect to acid value and 60 °C over 20 weeks (error bars are smaller than the size of the symbols).

The acid value (AV) of pure *N. cataria* oil reaches 4% in 7 days, while it increases much slower for the emulsion. The AV of the *N. cataria* Pickering emulsion increases the most in the presence of both light and heat. The AV of the reference sample rises 4-fold in 140 days (Fig. 6). It increases 6-fold in 140 days for the emulsion exposed to either heat or light and 9-fold in 3 weeks for the sample exposed to light and heat. For an ester terpenoid such as nepetalactone, organic acids are either the products of ester hydrolysis, or the secondary oxidation products. Rajeswara Rao *et al*.^53^ did not report significant physicochemical changes in essential oils stored in water, even at 20% (v/v). Therefore, double bond oxidation is the most probable pathway to organic acids, confirmed by a concomitant decrease in the IV.

### Acceptability study

During a one-month trial, the majority of the participants (52/60) applied the lotion correctly while 7 individuals applied the lotion sporadically: 5 individuals applied the lotion 3 times before sleeping, 40 applied it 2 times and 15 applied it only once. In general, most of the individuals (91.6%) apply lotions after a shower to keep skin hydrated while 5 men do not routinely apply lotions. Among the adolescents and adults (31 participants), most found the odor tolerable while 10 stated that it was strong smelling (pungent). The side effects (Table 2) included sneezing, feeling nauseous, and vomiting, but only 6 individuals reported these effects.

**Table 2:** Reported side effects.

Undesirable Effects	Individuals, n = 60
Sneezing	3
Nausea	2
Vomiting	1
Total	6

Almost everyone agreed that the lotion reduced how often they were bitten. Only one individual said that it was ineffective and that they were bitten as often with the repellent; 55 individuals said that they were not bitten after applying the lotion (Table 3). Here we assign a value of 2 to individuals finding the essential oil effect effective and 1 to those who did not. Assuming the responses are normally distributed, the p-value was 0.001 for a null hypothesis that the essential oil was ineffective, which allows us to conclude that it was effective.

**Table 3:** Lotion effectiveness according to the survey.

Comments	Individuals, n = 60
Ineffective	1
Lotion reduces bites	4
No bites after application	55

## Discussion

To introduce NPL as a strategy to reduce mosquito bites requires governmental approval from several ministries’ (Health, Agriculture, Science and Education, Planning and Reconstruction), and collaborations from university (faculty of medicine, agriculture), UN agencies (WHO, Global Fund, UNICEF), foreign donors, governments, and agricultural cooperatives who devised the implementation strategy. Working with the media acquaints the population to the opportunities and challenges and plays an important role mobilizing the decision makers at the local and political sphere.

Applying the mosquito repellent frequently demonstrates a high degree of acceptability. Rather than just lotions, the 31 individuals from the study also suggested incorporating the essential oil in perfumes (21), soaps (25), and indoor sprays (20). Rowland *et al*.^54^ reported that adding DEET to soap reduced the number of cases of malaria by 50%.

These results are encouraging considering that, on average, people may be bitten 75 times per night^55^. The combination of mosquito repellents and insecticide impregnated nets reduced malaria 80% more than for individuals that only slept under nets^56^.

Mosquitos are developing resistance to insecticide treated nets and they are changing their feeding habits to adapt to the reduced availability of their prey during the night. These adaptations by the *Anopheles* mosquito requires new strategies to ensure that the world malaria death rates continue to decline. We recommend that local populations grow and produce their own mosquito repellent with sustainable resources like coffee husks, bagasse, or other waste lignocellulosic feeds stocks. More work is required to identify alternative energy sources and extraction technologies that are more efficient to isolate the essential oil. Furthermore, we recognize that mosquitos may also develop a tolerance for any repellent and therefore, we must continue to develop other agriculturally based compounds to protect vulnerable populations, particularly children and pregnant women.

## Methods

### Steam distillation

KarireProducts cultivated *N. cataria* in the Moso region in the province of RUTANA, Burundi, picked the leaves at full bloom, and air-dried them for 30 hours. In a 5 hour process, a stainless steel steam distillation system isolated essential oil from chopped dried plant material (20 to 25 kg/batch). We stored the essential oil in amber containers at low temperature (4 °C). A propane burner boiled water at the bottom of a tank and the steam it generated passed through a metal mesh that was supporting the weight of the leaves. The steam first heats the biomass. After one hour, both oil and water vapour broke through to the top of the distillation column and passed through a pipe at the top lead to a condenser. The condensed solution separated in a second vessel and we collected both the hydrosol and essential oil.

### Ultrasound extraction

A Sonics Vibracell Ultrasound probe delivered 20 W (500 W nominal power) to 20 mL of water and ethanol in which we added 1 leaf (approximately 0.2 g) of catnip collected before the plant flowered. Sonication lasted 1 min. A thermocouple measured the temperature of the liquid. We collected all samples in 2 mL glass vials. The reference samples were stored in a 500 mL hermetically sealed dark brown glass bottle that we covered in aluminum foil and stored in a chemical cabinet maintained at ambient temperature.

### Scanning electron microscopy

A field emission scanning electron microscope (FE-SEM-JEOL JSM-7600F) with a voltage of 2 kV imaged catmint leaves (LEI detector). The distance between sample and detector was 13 mm.

### Gas chromatography

*N. cataria* oil is a clear pale yellow liquid, with a refraction index of 1.4882 ± 0.0005 (23 °C). We diluted the samples in HPLC grade pentane (1:200). A gas chromatograph equipped with a flame ionisation detector (GC-FID) and another GC coupled to a mass spectrometer (GC-MS) quantified the main components of the essential oil. The GC-FID was equipped with a DB-5 column (30 m × 0.25 mm × 0.25 m; Agilent Technologies, Santa Clara, CA, USA), while the GC-MS had a Solgel-Wax column (30 m × 0.25 mm × 0.25 m; SGE Analytical Science, Austin, TX, USA). We set both GCs injection port and detector temperature at 220 °C and 260 °C, respectively. Helium carried analytes at a flow rate of 1.4 mL/minute. Temperature remained at 40 °C for 2 minutes, then increased to 210 °C at a rate of 2 °C/minute. The split ratio was 50:1 and the injection volume was 3 microlites. The mass spectrometer operated in electron-impact (EI) mode at 70 eV, with a scan range of 40–550 amu and a scan speed of 1458.6 amu/s. The temperature of the MS interface was 300 °C. We computed FID peak areas without a correction factor. The retention times of n-alkanes with an even number of hydrocarbons (C8–C24) injected using the same analytical conditions determined the retention indices of the essential oil constituents. We qualitatively identified all the compounds from the NIST library^57^. To identify the geometrical isomers we injected standards, purchased from Sigma Aldrich.

### Stability tests

For the light stability tests we exposed the samples to a UV-A lamp (Spectroline EA-160), while for the heat stability we placed the samples in an oven at 60 ± 3 °C. In the combined light and heat stability tests we placed the oil containing vials at the bottom of a thermic bath (ISOTEMP 205) at 60 ± 3 °C surmounted with the UV-A lamp.

We applied ASTM-D5768 to measure the iodine value of the essential oil, which represents the degree of unsaturation: the iodine reacts with the double bonds, also known as the Wijs procedure.

We reported the acid value as g of KOH required to neutralize 100 g of sample.

### Acceptability study

After meeting with presidential staff and ministers of health and agriculture, students in the faculty of medicine developed a strategy to assess the population’s acceptability of applying mosquito repellents daily. We produced a lotion that contained a mixture of 5% nepetalactone in vegetable oil, almond oil and citronella. In 2011 from March 3^rd^ to April 3^rd^, the students followed 8 families in Kamenge (an urban neighborhood of Bujumbura) as selected by the non-governmental agency ALUMA respecting pre-established selection criteria and 4 families in the rural community of Cibitoke (selected by the local hospital). Both were hyper endemic malaria regions. Each family received 2 bottles with 100 mL of the lotion. The volunteers included one pregnant woman, 10 children younger than 5 y, 19 children from 5 to 11 y, 11 adolescents from 12 to 20 y, and 20 adults older than 20 y of which most of these were farmers (17). There were 32 males and 28 females in the study. Most individuals (45%) were in bed under impregnated mosquito nets by 21h00, while 32% were in bed by 22h00, and 10 children were in bed before 20h00.

### Data availability

All data generated or analyzed during this study are included in this published article (and its Supplementary Information files).

### Ethics statement

All participants and guardians were enrolled from March 3^rd^ to April 3^rd^ 2011 and provided written informed consent. Action de Lutte contre la Malaria au Burundi (ALUMA) and the hospital of Cibitoke selected the individuals. The hospital of Cibitoke and ALUMA approved the experimental protocol and it was carried out in accordance to all relevant guidelines and regulations.

## Electronic supplementary material

Supplementary Information

## References

[1] World Health Organization. World malaria report 2015. ISBN: 978 92 4 156515 8 (2015).

[2] Havyarimana, M. Burundi declares malaria epidemic. *Science and Health* http://www.theeastafrican.co.ke/scienceandhealth/Burundi-declares-malaria-epidemic/3073694-3848428-ov069/index.html (2017).

[3] Sachs JD, Warner AM (1997). Sources of slow growth in African economies. J. Afr. Econ..

[4] Brennan, K. Burundi: the evolution of Africa’s major nations. (ed. Brennan, K.) 1–79 (Mason Crest, 2013).

[5] Soto-Calle V (2017). Spatio-temporal analysis of malaria incidence in the Peruvian Amazon region between 2002 and 2013. Sci. Rep..

[6] Noedl H (2008). Evidence of artemisinin-resistant malaria in western Cambodia. N. Engl. J. Med..

[7] O’Neill PM (2017). A tetraoxane-based antimalarial drug candidate that overcomes PfK13-C580Y dependent artemisinin resistance. Nat. Commun..

[8] Krishna S, Bustamante L, Haynes RK, Staines HM (2008). Artemisinins: their growing importance in medicine. Trends Pharmacol Sci..

[9] Burke A (2017). A new malaria vector mosquito in South Africa. Sci. Rep..

[10] Gamble C, Ekwaru J, ter Kuile F (2009). Insecticide-treated nets for preventing malaria in pregnancy (Review). Cochrane Libr..

[11] Kamaraj C (2009). Larvicidal potential of medicinal plant extracts against Anopheles subpictus Grassi and Culex tritaeniorhynchus Giles (Diptera: Culicidae). Parasitol. Res..

[12] Ikeda T (2017). Seasonally lagged effects of climatic factors on malaria incidence in South Africa. Sci. Rep..

[13] Graves P, Gelband H (2006). Vaccines for preventing malaria (blood-stage). Cochrane Database Syst. Rev..

[14] Hutton G, Tediosi F (2006). The costs of introducing a malaria vaccine through the expanded program on immunization in Tanzania. Am. J. Trop. Med. Hyg..

[15] Moiroux N (2012). Changes in *Anopheles funestus* biting behavior following universal coverage of long-lasting insecticidal nets in Benin. J. Infect. Dis..

[16] Wamae PM, Githeko AK, Otieno GO, Kabiru EW, Duombia SO (2015). Early biting of the Anopheles gambiae s.s. and its challenges to vector control using insecticide treated nets in western Kenya highlands. Acta Trop..

[17] Katz TM, Miller JH, Hebert AA (2008). Insect repellents: historical perspectives and new developments. J. Am. Acad. Dermatol..

[18] Isman MB (2006). Botanical insecticides, deterrents, and repellents in modern agriculture and an increasingly regulated world. Annu. Rev. Entomol..

[19] Fradin MS, Day JF (2002). Comparative efficacy of insect repellents against mosquito bites. N. Engl. J. Med..

[20] Syed Z, Leal WS (2008). Mosquitoes smell and avoid the insect repellent DEET. Proceedings of the National Academy of Sciences..

[21] Odalo JO (2005). Repellency of essential oils of some plants from the Kenyan coast against Anopheles gambiae. Acta Trop..

[22] USDA. Nepeta cataria L. catnip. https://plants.usda.gov/core/profile?symbol=NECA2 (2001)

[23] Duke, J. A. *Handbook of medicinal herbs*. (ed. Duke, J. A.) 1–896 (CRC press, 2002).

[24] Akhshi, S., Shafaghat, A. & Salehzadeh, J. Chemical composition and antibacterial activity of the essential oil the leaf of Nepeta persica. *Leonardo J. Sci*. 131–138 (2014).

[25] Zomorodian, K. *et al*. Chemical composition and antimicrobial activities of essential oils from Nepeta cataria L. against common causes of food-borne infections. *ISRN Pharm*. **1**, 10.5402/2012/591953 (2012). 10.5402/2012/591953 PMC3385634 22779012

[26] Patel EK, Gupta A, Oswal RJ (2012). A review on mosquito repellent methods. Int. J. Pharm. Chem. Biol. Sci..

[27] Zhu JJ (2009). Efficacy and safety of catnip (Nepeta cataria) as a novel filth fly repellent. Med. Vet. Entomol..

[28] Zhu JJ (2014). Efficacy and longevity of newly developed catnip oil microcapsules against stable fly oviposition and larval growth. Med. Vet. Entomol..

[29] Schreck CE (1977). Techniques for the evaluation of insect repellents: a critical review. Ann. Rev. Entomol..

[30] Sanghong R (2015). Remarkable repellency of Ligusticum sinense (Umbelliferae), a herbal alternative against laboratory populations of Anopheles minimus and Aedes aegypti (Diptera: Culicidae). Malar. J..

[31] Bernier UR, Furman KD, Kline DL, Allan SA, Barnard DR (2005). Comparison of contact and spatial repellency of catnip oil and N,N-diethyl-3-methylbenzamide (deet) against mosquitoes. J. Med. Entomol..

[32] Feaster JA (2009). Dihydronepetalactones deter feeding activity by mosquitoes, stable flies, and deer ticks. J. Med. Entomol..

[33] Amer A, Mehlhorn H (2006). Repellency effect of forty-one essential oils against Aedes, Anopheles, and Culex mosquitoes. Parasitol. Res..

[34] Small E (2012). Catnip – safer pesticide potential. Biodiversity.

[35] Dadzie S (2013). A community-wide study of malaria reduction: evaluating efficacy and user-acceptance of a low-cost repellent in northern Ghana. Am. J. Trop. Med. Hyg..

[36] Mng’ong’o FC (2011). Repellent Plants Provide Affordable Natural Screening to Prevent Mosquito House Entry in Tropical Rural Settings — Results from a Pilot Efficacy Study. PLoS One..

[37] Birkett MA, Pickett JA (2003). Aphid sex pheromones: from discovery to commercial production. Phytochemistry..

[38] Kaya A, Demirci B, Baser KHC (2007). Micromorphology of glandular trichomes of Nepeta congesta Fisch. & Mey. var. congesta (Lamiaceae) and chemical analysis of the essential oils. South African J. Bot..

[39] Park, C. *et al*. Catnip as a source of essential oils. In: Creating markets for economic development of new crops and new uses (ed. Whipkey, A.) 311–315 (ASHS press, 2007).

[40] Mohammadi S, Saharkhiz MJ (2011). Changes in essential oil content and composition of catnip (Nepeta cataria L.) during different developmental stages. J.Essent.Oil Bear. Plants..

[41] Asgarpanah J, Sarabian S, Ziarati P (2014). Essential oil of Nepeta genus (Lamiaceae) from Iran: a review. J. Essent. Oil Res..

[42] Chalchat JC, Lamy J (1997). Chemical composition of the essential oil Isolated from wild Catnip Nepeta cataria L. cv. citriodora from the Drôme Region of France. J. Essent. Oil Res..

[43] Gilani AH (2009). Chemical composition and mechanisms underlying the spasmolytic and bronchodilatory properties of the essential oil of Nepeta cataria L. J. Ethnopharmacol..

[44] Werther J, Saenger M, Hartge EU, Ogada T, Siagi Z (2000). Combustion of agricultural residues. Prog. Energy Combust. Sci..

[45] McKendry P (2002). Energy production from biomass (part 1): Overview of biomass. Bioresource Technology.

[46] Gilbert P, Ryu C, Sharifi V, Swithenbank J (2009). Effect of process parameters on pelletisation of herbaceous crops. Fuel.

[47] International Association for Medical Assistance to Travellers. Climate information for Bujumbura Burundi, https://www.iamat.org/country/burundi/climate-data#null (2017)

[48] Turek C, Stintzing FC (2013). Stability of essential oils: A review. Comprehensive Reviews in Food Science and Food Safety.

[49] Mcgraw GW, Hemingway RW, Ingram LL, Canady CS, Mcgraw WB (1999). Thermal degradation of terpenes: Camphene,  Δ^3^-carene, limonene, and  α-terpinene. Environ. Sci. Technol..

[50] de Barros Fernandes RV (2016). Study of ultrasound-assisted emulsions on microencapsulation of ginger essential oil by spray drying. Ind. Crops Prod..

[51] Boffito DC, Pirola C, Galli F, Di Michele A, Bianchi CL (2013). Free fatty acids esterification of waste cooking oil and its mixtures with rapeseed oil and diesel. Fuel..

[52] Guenther, E. In The essential oils Vol. I: History – origin in plants – production – analysis (ed. Guenther, E.) 377–379 (Read Books Ltd, 2013).

[53] Rajeswara Rao BR, Rajput DK, Patel RP (2011). Storage of essential oils: Influence of presence of presence of water for short periods on the composition of major constituents of the essential oils of four economically important aromatic crops. J. Essent. Oil Bear. Plants..

[54] Rowland M (2004). DEET mosquito repellent provides personal protection against malaria: A household randomized trial in an Afghan refugee camp in Pakistan. Trop. Med. Int. Heal..

[55] Van Bortel W, Barutwanayo M, Delacollette C, Coosemans M (1996). Motivation to acquire and use impregnated mosquito nets in a stable malaria zone in Burundi. Trop. Med. Int. Heal..

[56] Hill N, Lenglet A, Arnez AM, Carneiro I (2007). Plant based insect repellent and insecticide treated bed nets to protect against malaria in areas of early evening biting vectors: double blind randomised placebo controlled clinical trial in the Bolivian Amazon. BMJ..

[57] Sparkman OD (2005). Identification of essential oil components by gas chromatography/quadrupole mass spectroscopy Robert P. Adams. J. Am. Soc. Mass Spectrom..

[58] Regnier FE, Waller GR, Eisenbraun EJ (1967). Studies on the composition of the essential oils of three Nepeta species. Phytochemistry..

[59] Bourrel C, Perineau F, Michel G, Bessiere JM (1993). Catnip (Nepeta cataria L.) Essential oil: analysis of chemical constituents, bacteriostatic and fungistatic properties. J. Essent. Oil Res..

[60] Malizia RA, Molli JS, Cardell DA, Retamar JA (1996). Volatile constituents of the essential oil of Nepeta cataria L. grown in Cordoba province (Argentina). J. Essent. Oil Res..

[61] Handjieva NV, Popov SS, Evstatieva LN (1996). Constituents of essential oils from Nepeta cataria L., N. grandiflora M.B. and N. nuda L. J. Essent. Oil Res..

[62] Schultz G, Simbro E, Belden J, Zhu J, Coats J (2004). Catnip, Nepeta cataria (Lamiales: Lamiaceae)—A closer look: seasonal occurrence of Nepetalactone isomers and comparative repellency of three terpenoids to insects. Environ. Entomol..

[63] Morteza-Semnani K, Saeedi M (2004). Essential oils composition of Nepeta cataria L. and Nepeta crassifolia Boiss. and Buhse from Iran. J. Essent. Oil Bear. Plants..

[64] Adiguzel A (2009). Antimicrobial and antioxidant activity of the essential oil and methanol extract of Nepeta cataria. Polish J. Microbiol..

[65] Birkett MA, Hassanali A, Hoglund S, Pettersson J, Pickett JA (2011). Repellent activity of catmint, Nepeta cataria, and iridoid nepetalactone isomers against Afro-tropical mosquitoes, ixodid ticks and red poultry mites. Phytochemistry..

[66] Srifi A (2013). Étude phytochimique et activité antifongique *in vitro* des huiles essentielles de quatre espèces du genre Nepeta du Maroc. Phytothérapie..

[67] Kilic O, Behcet L, Bagci E (2013). Essential oil compounds of three Nepeta L. Taxa from Turkey and their chemotaxonomy. Asian J. Chem..

