# Supplementary material for: Sustainable manufacture of insect repellents derived from Nepeta cataria
Source: Sci Rep. 2018 Feb 2;8:2235. doi: 10.1038/s41598-017-18141-z (PMC5797122; doi:10.1038/s41598-017-18141-z)
Supplement: Supplementary file 1 — Supplementary Information [file 41598_2017_18141_MOESM1_ESM.doc]

**Sustainable manufacture of insect repellents derived from *Nepeta Cataria***

Gregory S. Patience^1*^, Ginette Karirekinyana^2^ Federico Galli^1^, Nicolas A. Patience^1^, Cariton Kubwabo^2^, Guy Collin^3^, Jean Claude Bizimana, Daria C. Boffito^1^

^1^Département de Génie Chimique, École Polytechnique Montréal, 2900, boul. Édouard-Montpetit, Montréal (Qc), Canada H3C 3A7

^2^De l’Agence consultative en éthique de coopération internationale (ACECI), 11 Rue Mugamba, Quartier Rohero II, Bujumbura, Burundi

^3^Département des sciences fondamentales, UQAC, Saguenay (Qc), Canada G7H 2B1

*Corresponding author: Gregory S. Patience

E-mail: [gregory-s.patience@polymtl.ca](mailto:gregory-s.patience@polymtl.ca)

Telephone: +1 (514) 340-4711 x 3439

**Table S1.** Chemical composition of the essential oil from aerial parts of *N. cataria* in Burundi

| **Compound** | | **Structure** | | **DB-5** | | | | | **Solgel-Wax** | |
| --- | --- | --- | --- | --- | --- | --- | --- | --- | --- | --- |
|  |  |  |  | Retention time (min) | | Retention index | | Area (%) | Retention index | |
| 5-Methyl-3-hexen-2-one | | 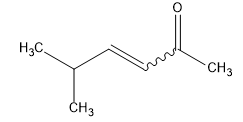 | | 9.88 | | 872 | | <0.05 | 1230 | |
| α-Pinene | | 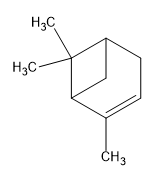 | | 12.23 | | 918 | | <0.05 | 1005 | |
| Sabinene | | 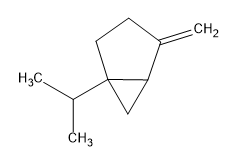 | | 14.59 | | 963 | | 0.1 | 1085 | |
| β-Pinene | | 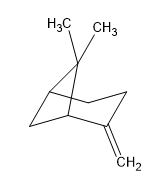 | | 14.70 | | 966 | | 0.3 | 1070 | |
| Oct-1-en-3-ol | | 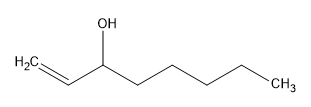 | | 15.15 | | 974 | | 0.1 | 1433 | |
| 6-Methyl-5-hepten-2-one? | | 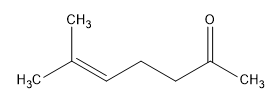 | | 15.49 | | 980 | | <0.05 |  | |
| Myrcene | | 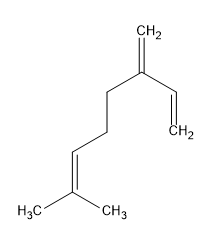 | | 15.84 | | 987 | | <0.05 | 1138 | |
| Limonene | | 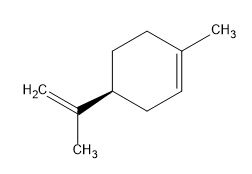 | | 18.09 | | 1024* | | <0.05 | 1172 | |
| β-Phellandrene | | 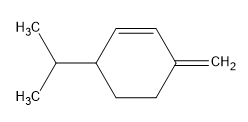 | | 18.09 | | 1024* | | <0.05 | 1180 | |
| ***cis*-β-Ocimene** | | 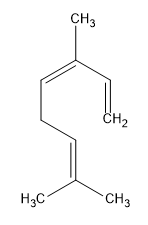 | | 18.90 | | 1036 | | 1.2 | 1219 | |
| ***trans*-β-Ocimene** | | 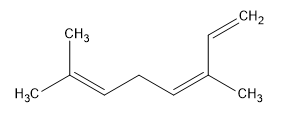 | | 19.57 | | 1046 | | 3.8 | 1233 | |
| Linalool | | 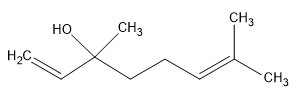 | | 23.02 | | 1097 | | <0.05 | 1530 | |
| Nonanal | | 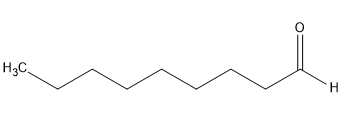 | | 23.27 | | 1100 | | <0.05 | 1372 | |
| Terpinen-4-ol | | 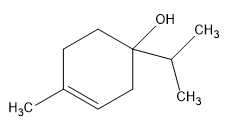 | | 28.04 | | 1171 | | <0.05 | 1569 | |
| Methyl salicylate | | 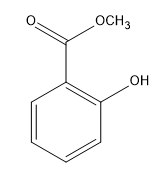 | | 29.07 | | 1186 | | 0.2 | 1723* | |
| **4aα,7α,7aα-Nepetalactone** | | 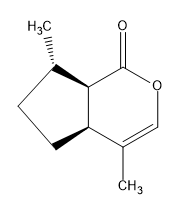 | | 40.05 | | 1356 | | 72.1 | 1909 | |
| Carvacrol acetate | | 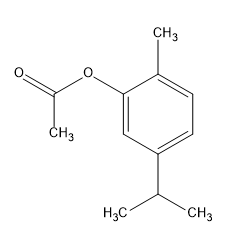 | | 41.04 | | 1371 | | <0.05 | 1842 | |
| **4aα,7α,7aβ-Nepetalactone** | | 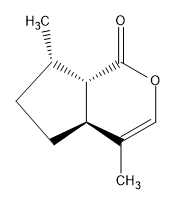 | | 41.54 | | 1379 | | 0.5 | 1956 | |
| **4aα,7β,7aα-Nepetalactone** | | 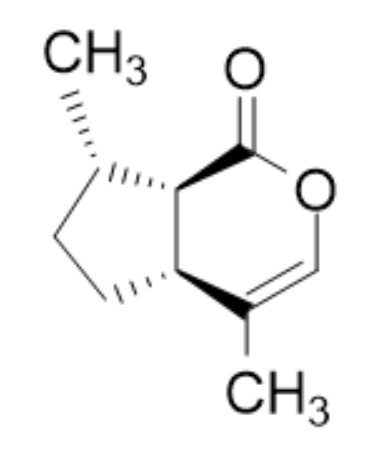 | | 41.90 | | 1384 | | 1.1 | 1975 | |
| Dihydronepetalactone | | 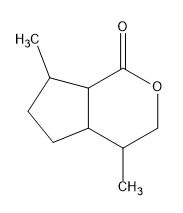 | | 12.98 | | 1404 | | 0.4 | 2032 | |
| **β-Caryopyllene** | | 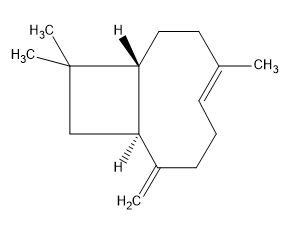 | | 43.64 | | 1412 | | 10.1 | 1558 | |
| α-Humulene | | 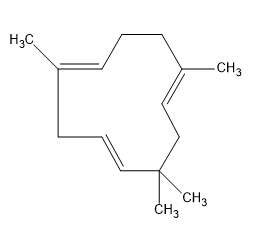 | | 45.66 | | 1447 | | 0.8 | 1625 | |
| *trans*-β-Farnesene | | 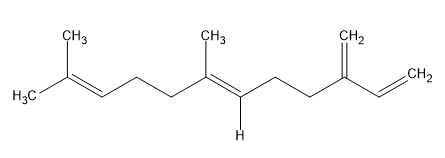 | | 46.19 | | 1456 | | <0.05 | 1645 | |
| Nepetalic acid isomer? | | 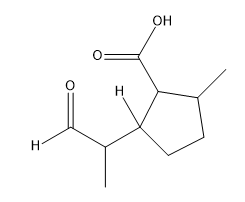 | | 48.56 | | 1496 | | 1.7 | - | |
| Germacrene A | | 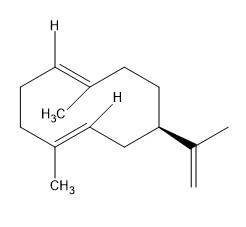 | | 48.72 | | 1499 | | 0.1 | 1737 | |
| (*E,E*)-α-Farnesene | | 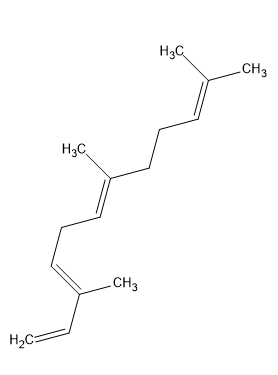 | | 49.23 | | 1508 | | 0.1 | 1723* | |
| Nepetalic acid isomer? | | 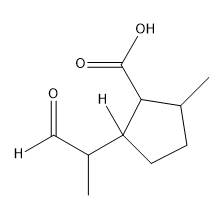 | | 51.10 | | 1540 | | 5.9 | 2828 | |
| Selina-3,7(11)-diene | | 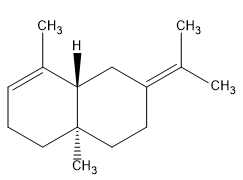 | | 51.35 | | 1544 | | <0.05 | 1750 | |
| Caryophyllene oxide | | 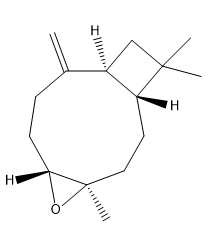 | | 53.07 | | 1574 | | 0.7 | 1916 | |
| Humulene epoxide II | | 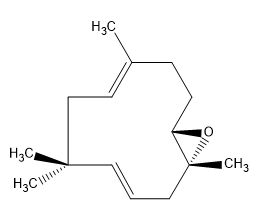 | | 54.54 | | 1599 | | <0.05 | 1995 | |
| **Total identified (%)** |  | |  | |  | | **99.2** | | |  |

Notes: ?, compound tentatively identified; *, two compounds co-eluting on the column.
